# Identification of a Genomic Instability-Related Long Noncoding RNA Prognostic Model in Colorectal Cancer Based on Bioinformatic Analysis

**DOI:** 10.1155/2022/4556585

**Published:** 2022-06-07

**Authors:** Yu Liang, Hong-Xu Sun, Bin Ma, Qing-Kai Meng

**Affiliations:** Department of Colorectal Surgery, Cancer Hospital of China Medical University, Liaoning Cancer Hospital & Institute, Shenyang, 110042 Liaoning Province, China

## Abstract

**Background:**

In recent years, a growing body of research has revealed that long noncoding RNAs (lncRNAs) participate in regulating genomic instability.

**Materials and Methods:**

We obtained RNA expression profiles, somatic mutation profiles, clinical information, and pathological features of colorectal cancer (CRC) from The Cancer Genome Atlas project. We divided the cohort into two groups based on mutation frequency and identified genomic instability-related lncRNAs (GI-lncRNAs) using R software. We further analyzed the function of identified GI-lncRNAs and established a prognostic model through Cox regression. Using the established prognostic model, we divided the cohort into the high- and low-risk groups and further verified the prognostic differences between the two groups as well as the predictive power of prognosis-related lncRNAs in the genomic instability of CRC.

**Results:**

We identified a total of 143 GI-lncRNAs that were differentially expressed between the higher mutation frequency group and the lower mutation frequency group. According to Kyoto Encyclopedia of Genes and Genomes pathway and Gene Ontology analyses, a series of cancer-associated terms were enriched. We further constructed a prognostic model that included five GI-lncRNAs (lncRNA PTPRD-AS1, lncRNA AC009237.14, lncRNA LINC00543, lncRNA AP003555.1, and lncRNA AL109615.3). We confirmed that the expression of the five GI-lncRNAs was associated with prognosis and the mutation of critical genes in the CRC patient cohort.

**Conclusions:**

The present research further confirmed the vital function of GI-lncRNAs in the genomic instability of CRC. The five GI-lncRNAs identified in our study are potential biomarkers and need to be studied in more depth.

## 1. Introduction

Colorectal cancer (CRC) is the most common malignant neoplasm of the digestive system. According to “Cancer Statistics, 2020” produced by the American Cancer Society, a total of 147,950 new cases and 53,200 deaths were registered from CRC in the United States [1]. Accumulating studies indicated that CRC is the result of a combination of environmental, dietary, lifestyle, and genetic factors [2]. A series of treatments including surgery, chemotherapy, targeted therapy, radiotherapy, and immunotherapy can improve patient outcomes; however, the prognosis of patients with CRC in the advanced stage is still poor [3]. Hence, there is an urgent need to explore and elucidate the molecular biological mechanisms and novel effective biomarkers like noncoding RNAs or circulating tumor DNA of CRC [4].

Genomic instability, including chromosomal instability and microsatellite instability (MSI), is an immensely complex molecular phenotype and mechanism [5]. Genomic instability has been verified as a “facilitating characteristic” that promotes cellular oncogenesis and metastasis [6]. Emerging evidence has indicated that inactivation of the mismatch repair system and the base excision repair system as well as genetic mutations is a critical mechanism in the tumorigenesis of CRC [7]. Long noncoding RNAs (lncRNAs) are a type of transcript of more than 200 nucleotides which do not encode proteins [8]. Recently, research into lncRNAs has been a new academic focus and attracted more interest. Accumulating evidence indicates that lncRNAs are involved in driving genomic instability at transcriptional and translational levels [9]. For instance, Lee et al. indicated that the lncRNA NORAD maintained ploidy and genomic stability by acting as a molecular decoy for PUMILIO proteins [10]. Chen et al. revealed that lncRNA CCAT2 drives chromosomal instability and carcinogenesis of CRC by upregulating the expression of the ribosomal biogenesis factor BOP1 [11].

With the development and utilization of next-generation sequencing platforms, an increasing number of novel genes and mutations have been identified and verified to play critical roles in tumor progression. For example, exome sequencing was utilized to identify CRC somatic mutations, and paternally expressed gene 3 (PEG3) was verified as a high-frequency mutated gene [12]. Imperial et al. performed comparative somatic and proteomic analyses of right-sided colon cancer, left-sided colon cancer, and rectal cancers. The results indicated that a nonsense mutation of adenomatous polyposis coli (APC) was a biomarker of right-sided colon cancer, and hub proteins in protein-protein interaction networks have critical roles in left- or right-sided colon cancer [13].

Recently, an accumulating body of bioinformatic studies was focused on the identification of potential biomarkers associated with tumor progression. For instance, seven identified miRNAs (miR-139-5p, miR-146a-5p, miR-185-5p, miR-195-5p, miR-340-5p, miR-331-3p, and miR-484) were found related to development, lifestyles, and overall survival of breast cancer patients [14]. And four differently expressed miRNAs (miR-21-5p, miR-183-5p, miR-195-5p, and miR-497-5p) were related to CRC through multiple signaling pathways based on the GEO datasets [15]. Lu et al. established a metabolism-related lncRNA prognostic model to predict the clinical outcome of CRC patients [16]. To the best of our knowledge, however, there are few academic researches relevant to lncRNAs and genomic instability of CRC based on bioinformatic analysis. In the present research, we obtained *in silico* data from The Cancer Genome Atlas (TCGA, http://cancergenome.nih.gov) project [17] and performed bioinformatic analysis to identify genomic instability-related lncRNAs (GI-lncRNAs). Furthermore, we constructed a prognostic model to predict the overall survival and critical genomic mutations of patients.

## 2. Materials and Methods

### 2.1. Data Collection and GI-lncRNA Identification

We downloaded transcriptome profiles (RNA-Seq), simple nucleotide variation (masked somatic mutation), and clinical data from TCGA database (https://portal.gdc.cancer.gov). The inclusion criteria were as follows: the samples (1) were pathologically diagnosed as colon adenocarcinoma or rectal adenocarcinoma, (2) had integral clinical and pathological data, and (3) had the follow-up time and surviving state. As a result, a total of 568 CRC and 44 normal tissues were included in this research. According to the documents and guidelines published by TCGA Authority (https://cancergenome.nih.gov/publications/publicationguidelines), the present research does not require ethical review.

We extracted the lncRNA expression matrix and genetic mutation information from TCGA samples using PERL software. The samples were then ranked according to the number of cumulative mutant genes. We further selected the top 25% and bottom 25% of the samples to compare differences in lncRNA expression. GI-lncRNAs were identified using the “edgeR” package in R software [18] with a threshold log2 fold change > 1.0 and *P* < 0.01. Furthermore, a heatmap that included the 20 top upregulated and 20 top downregulated GI-lncRNAs was mapped using the “heatmap” package in R software. According to the expression of GI-lncRNAs, we regrouped 568 samples into the genomically stable-like group (GS-like group) and a genomically unstable-like group (GU-like group) through clustering analysis. Genetic mutations and gene expressions between the two groups were also analyzed.

### 2.2. GI-lncRNA Enrichment Analyses

We performed Spearman's correlation analysis to identify the mRNAs that were coexpressed with GI-lncRNAs. Each lncRNA that corresponded to 10 mRNAs was shown in a network diagram. Moreover, we performed Gene Ontology (GO) [19] and Kyoto Encyclopedia of Genes and Genomes (KEGG) [20] pathway analyses using “clusterProfiler,” “org.Hs.eg.db,” “enrichplot,” and “ggplot2” packages in R software. The results of GO and KEGG analyses were visualized in a bar chart, and *P* < 0.05 was regarded as statistically significant.

### 2.3. Construction of a GI-lncRNA Prognostic Model

A total of 509 CRC patients, whose follow-up time was more than 30 days and for whom complete clinical data were available, were enrolled in this study. We randomly divided the samples into a “train” group and a “test” group, and then, we performed Cox proportional hazard regression analysis to identify the GI-lncRNA signature. We then identified GI-lncRNAs which were significantly related to survival in the “train” group and the “test” group (*P* < 0.05). Calculation of hazard ratio and 95% confidence interval and generation of the profile of the receiver operating characteristic (ROC) curve were performed by applying “survival,” “caret,” “glmnet,” “survminer,” and “timeROC” packages in R software.

We further identified the GI-lncRNA signature as an independent prognostic factor based on the coefficient in Cox multivariate analysis. The model that included the expression of the GI-lncRNA signature and coefficient was constructed as follows:

(1) $$\begin{array}{c} \text{Risk score}=\overset{n}{\underset{i=1}{\sum }}\text{C}\text{oefficient}\left(\text{GI}‐\text{lncRNA}i\right)\times \text{Expression}\left(\text{GI}‐\text{lncRNA}i\right). \end{array}$$

According to the risk score, the samples were further divided into a high-risk group and a low-risk group. The Kaplan-Meier method was utilized to compare overall survival between the two groups. We calculated the hazard ratios and 95% confidence intervals of age, TNM stage, and risk score. Furthermore, we analyzed differences in the mutation of critical genes in CRC between the two groups.

## 3. Results

### 3.1. Identification of GI-lncRNAs in CRC

**Figure 1:**
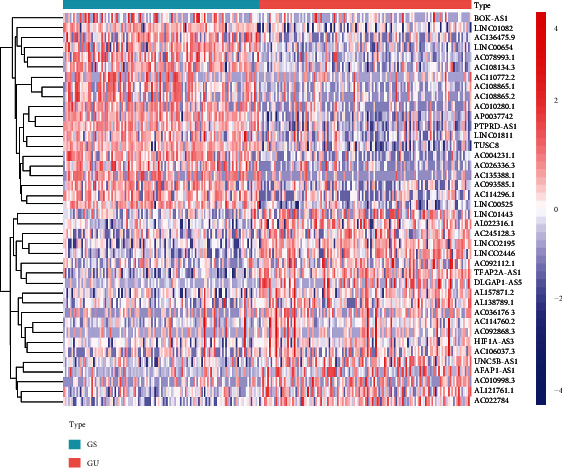
Heatmap of the top 40 genomic instability-related lncRNAs with the most significant differences in expression between the groups. The genomically stable (GS) group is shown below the green line, and the genomically unstable group is shown below the red line. The genomic instability-related lncRNA names are listed on the right vertical axis (log_2_ fold change > 1, *P* < 0.01).

To identify the GI-lncRNAs in CRC, we compared lncRNA expression between the GS samples (*n* = 118) and the GU samples (*n* = 128). A total of 143 GI-lncRNAs were identified which were significantly differentially expressed between the two groups. Among the GI-lncRNAs, 67 GI-lncRNAs were upregulated and 76 GI-lncRNAs were downregulated. The list of GI-lncRNAs is shown in Supplementary Materials, and the top 20 upregulated and downregulated GI-lncRNAs are shown as a heatmap in Figure 1.

**Figure 2:**
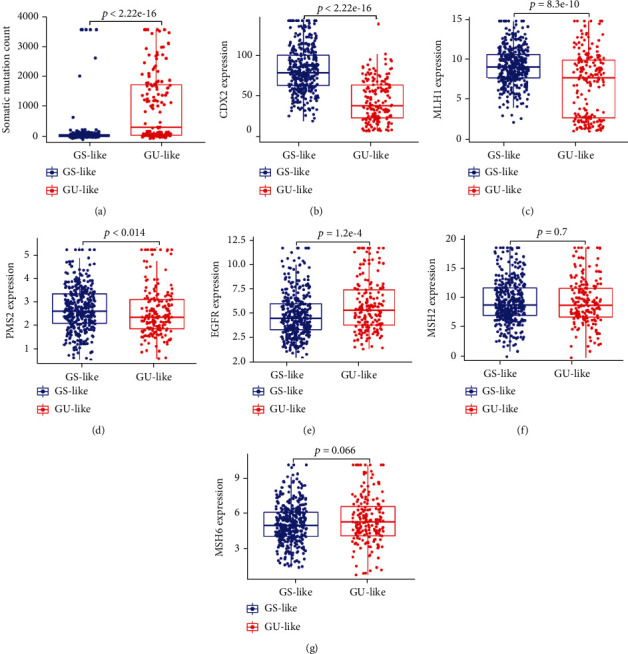
Analysis of differences in mutation and genetic expression between the genomically stable-like group and the genomically unstable-like group. (a) Somatic mutation count of genes in the genomically unstable-like group (GU-like) and the genomically stable-like group (GS-like group); (b–f) relative expression of genes between the GS-like group and the GU-like group; (b) CDX2; (c) MLH1; (d) PMS2; (e) EGFR; (f) MSH2G: MSH6. Data are shown as the mean ± SD.

We performed cluster detection to regroup the 568 samples into a GS-like group and a GU-like group. As a result, 363 samples were classified into the GS-like group, and 203 samples were classified into the GU-like group. The differential expression of GI-lncRNAs between the two groups is shown as a heatmap in Supplementary Figure 1. The number of mutant genes in the GU-like group was significantly more than that in the GS-like group (Figure 2(a), *P* < 2.22*e* − 16). Moreover, we analyzed differences in the expression of mismatch repair genes and colorectal oncogenes between the two groups. The expression of caudal-related homeobox transcription factor 2 (CDX2) (Figure 2(b), *P* < 2.22*e* − 16), mismatch repair gene mutL homolog 1 (MLH1) (Figure 2(c), *P* = 8.3*e* − 10), and postmeiotic segregation increased 2 (PMS2) (Figure 2(d), *P* = 0.014) was significantly lower in the GU-like group, while expression of epidermal growth factor receptor (EGFR) (Figure 2(e), *P* = 1.2*e* − 4) was higher in the GU-like group. However, there was no significant difference in the expression of mutator S homolog 2 (MSH2) (Figure 2(f), *P* = 0.7) or mutator S homolog 6 (MSH6) (Figure 2(g), *P* = 0.066) between the two groups.

### 3.2. Functional Enrichment Analyses of GI-lncRNAs

**Figure 3:**
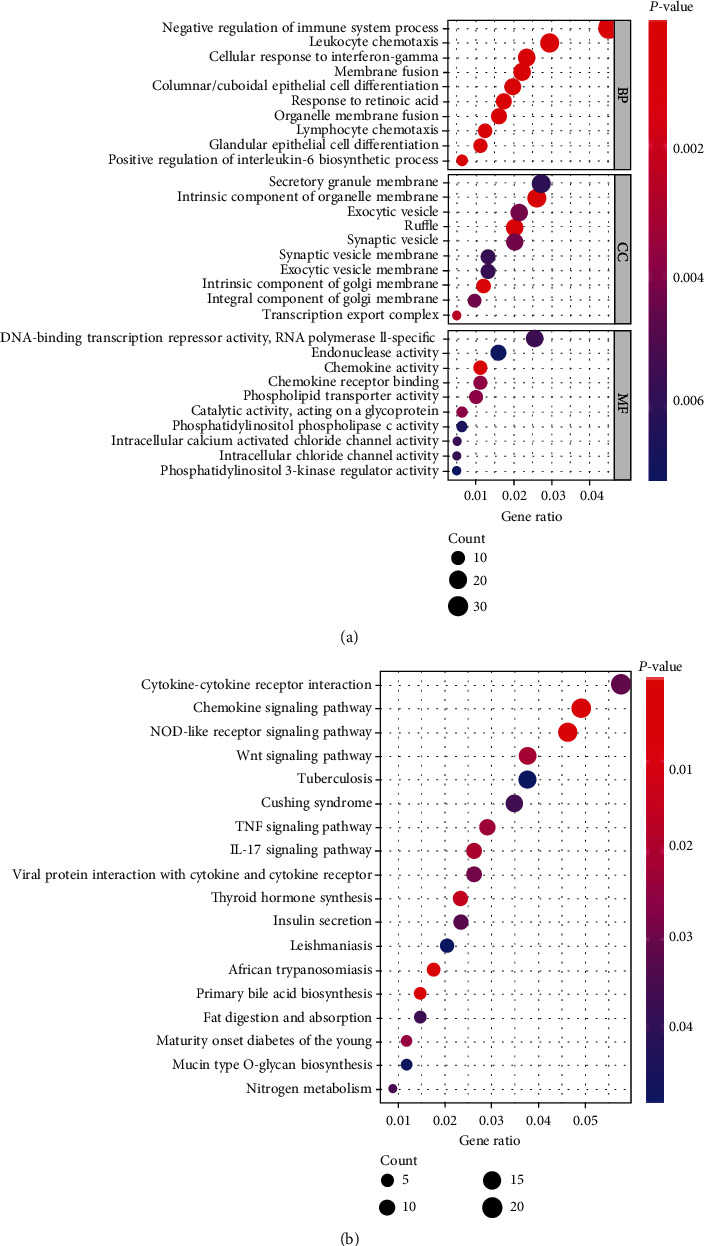
Gene Ontology and Kyoto Encyclopedia of Genes and Genomes Pathway enrichment analysis of coexpressed genes. (a) Biological process terms, cellular component terms, and molecular function terms of coexpressed genes; (b) Kyoto Encyclopedia of Genes and Genomes terms of coexpressed genes. The size of each “bubble” represents the number of genes.

To further explore the potential function of the 143 GI-lncRNAs, we performed GO and KEGG pathway analyses using R software. As shown in Supplementary Materials, we analyzed the 10 mRNAs with the highest coexpression coefficient with GI-lncRNAs. Moreover, we constructed the coexpression network that included the GI-lncRNAs and mRNAs (Supplementary Figure 2). The results of GO analysis indicated that the biological process and molecular function terms were mainly associated with immune-modulatory mechanisms and chemokine function, respectively (Figure 3(a)). The results of KEGG pathway analysis further indicated the involvement of multiple cancer-related pathways including “NOD-like (nucleotide-binding oligomerization domain-like) receptor signaling pathway,” “IL-17 (interleukin-17) signaling pathway,” “Wnt signaling pathway,” and “TNF (tumor necrosis factor) signaling pathway.” These results suggested that GI-lncRNAs may play critical roles in the progression of CRC (Figure 3(b)).

### 3.3. Construction of Prognostic Model Based on GI-lncRNAs

**Figure 4:**
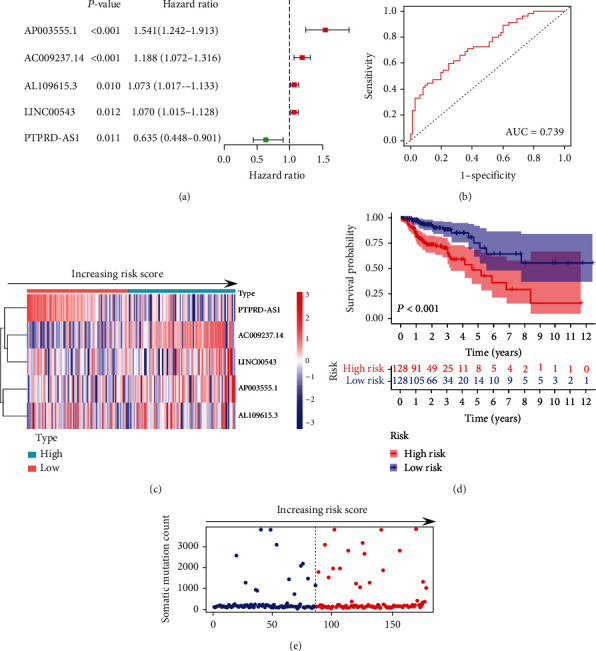
Identification of genomic instability-related lncRNA signature based on the “train” set. (a) Univariate analysis of five-genomic instability-related lncRNA (GI-lncRNA) signature in the “train” set; (b) ROC curve analysis of the five-GI-lncRNA signature in the “train” set; (c) the expression of the five-GI-lncRNA signature by increasing risk score in the “train” set, the median value of the risk score is taken as the cutoff, and samples are divided into the “high-risk” and “low-risk” groups; (d) Kaplan-Meier curve analysis of survival between the “high-risk” group and the “low-risk” group in the “train” set; (e) somatic mutation count by increasing risk score in the “train” set.

To further analyze the function of the GI-lncRNAs in predicting overall survival, we divided 509 CRC samples into the “train” and “test” groups; the clinicopathological characteristics between the two groups were not significantly different (Table 1). Then, we performed Cox proportional hazard regression analysis to identify the GI-lncRNA signature using the “train” group. As shown in Figure 4(a), we identified five GI-lncRNAs (lncRNA PTPRD-AS1, lncRNA AC009237.14, lncRNA LINC00543, lncRNA AP003555.1, and lncRNA AL109615.3) as independent prognostic factors using “train” group data. As shown in Figure 4(b), the area under the curve (AUC) of the ROC curve was 0.739. We further conducted Cox regression analyses to evaluate the prognostic role of the five-GI-lncRNA signature. The results, shown in Table 2, identified the five GI-lncRNAs as independent prognostic factors. According to the coefficient in multivariate analysis and expression of the five-GI-lncRNA signature, we constructed a prognostic model: risk score = (0.469 × AP003555.1 expression) + (0.182 × AC009237.14) + (0.072 × AL109615.3 expression) + (0.063 × LINC00543 expression) + (−0.447 × PTPRD‐AS1 expression). Based on the prognostic model, we then calculated the risk score of each sample and further plotted the heatmap by an ascending risk degree (Figure 4(c)). This provided preliminary evidence that PTPRD-AS1 is a protective factor and AC009237.14, LINC00543, AP003555.1, and AP003555.1 are risk factors. Furthermore, the low-risk samples exhibited better overall survival than the high-risk samples (Figure 4(d), *P* < 0.001). Moreover, with the increasing risk score, tumor somatic mutation count also increased, especially ranking between 50 and 150 (Figure 4(e)).

**Figure 5:**
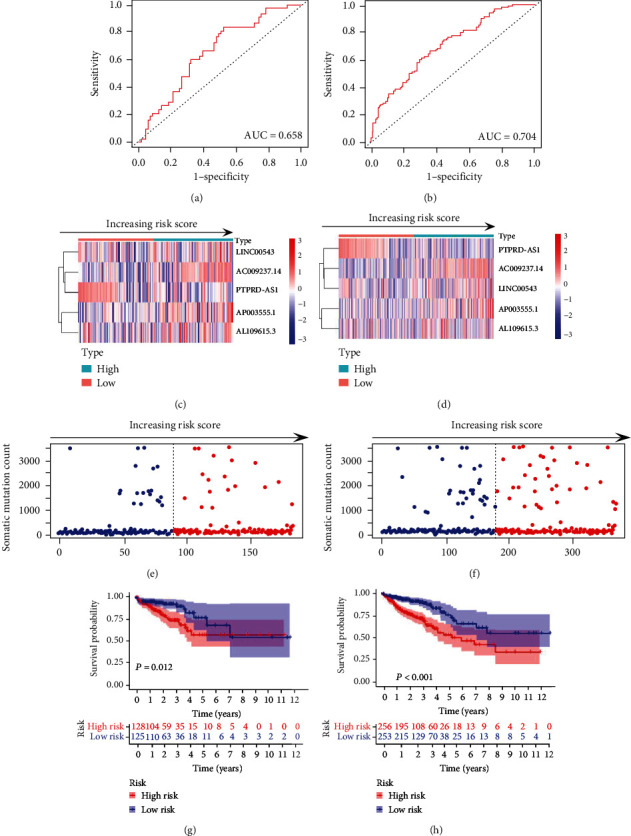
Validation of the five-genomic instability-related lncRNA (GI-lncRNA) signature. (a, b) ROC curve analysis of the five-genomic instability-related lncRNA (GI-lncRNA) signature in the “test” set and The Cancer Genome Atlas (TCGA) set; (c, d) expression of the five-GI-lncRNA signature by increasing risk score in the “test” set and TCGA set. The median value of risk score is taken as the cutoff, and samples were divided into the “high-risk” and “low-risk” groups; (e, f) somatic mutation count by increasing risk score in the “test” set and TCGA set; (g, h) Kaplan-Meier curve analysis of survival between the “high-risk” and “low-risk” groups in the “test” set and TCGA set.

We further utilized the “test” group samples and all TCGA set samples to verify the accuracy and reliability of the GI-lncRNA signature. The ROC curve analysis of the “test” set and TCGA set yielded AUCs of 0.658 and 0.704, respectively (Figures 5(a) and 5(b)). We also plotted the heatmap and ranked the risk score which was based on expression of the five GI-lncRNAs. The same as the results of the “train” group, PTPRD-AS1 was also verified as a protective factor, and AC009237.14, LINC00543, AP003555.1, and AP003555.1 were verified as risk factors (Figures 5(c) and 5(d)). We further analyzed the somatic mutation count in the “test” group and TCGA set, and the results indicated that samples in the quadrate range on each side of the median risk score had higher frequencies of mutation (Figures 5(e) and 5(f)). Kaplan-Meier analyses indicated that the low-risk group had better overall survival (Figure 5(g), *P* = 0.012, and Figure 5(h), *P* < 0.001). The above results confirmed the consistency and robustness of our model.

**Figure 6:**
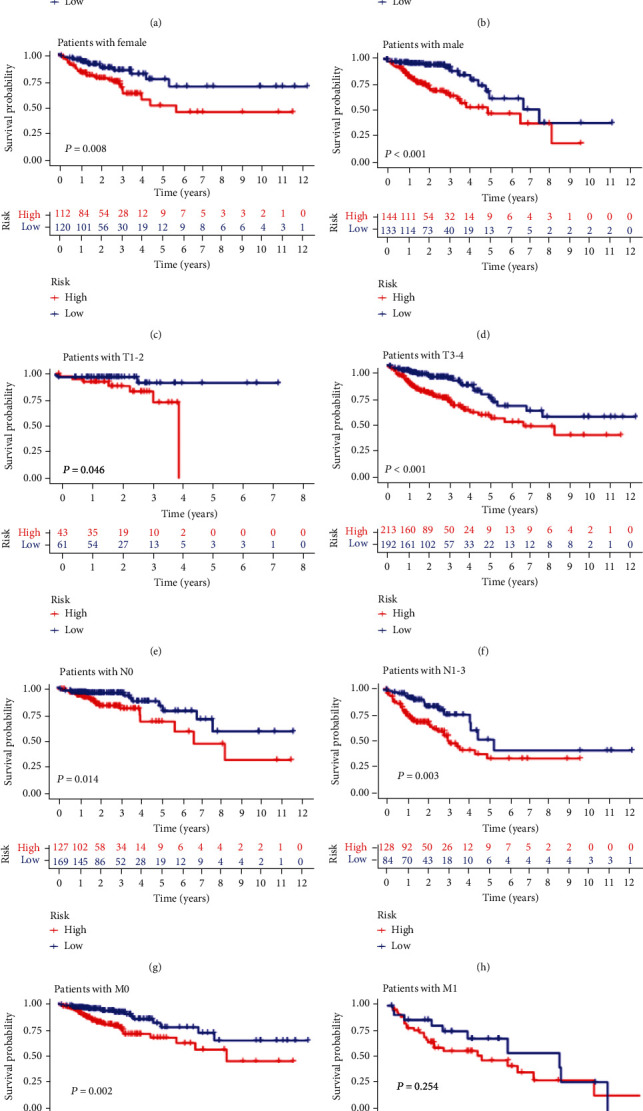
Survival analysis of the genomic instability-related lncRNA signature in relation to different clinical characteristics. (a, b) Kaplan-Meier curve analysis of survival between the “high-risk” and “low-risk” groups in the “>65 years old” set and the “≤65 years old” set; (c, d) Kaplan-Meier curve analysis of survival between the “high-risk” and “low-risk” groups in females and males; (e, f) Kaplan-Meier curve analysis of survival between the “high-risk” and “low-risk” groups in the T1-2 and T3-4 sets; (g, h) Kaplan-Meier curve analysis of survival between the “high-risk” and “low-risk” groups in the N0 and N1-3 sets; (i, j) Kaplan-Meier curve analysis of survival between the “high-risk” and “low-risk” groups in the M0 and M1 sets.

We next calculated the hazard ratio and 95% confidence interval of age, TNM stage, and risk score in TCGA set. As shown in Table 3, age, pTNM stage, and risk score were observed to be significant factors in both univariate and multivariate analyses. Then, we performed Kaplan-Meier curve analysis by age, pTNM stage, and gender to determine whether the GI-lncRNA signature was consistent across different pathological characteristics. The CRC samples of TCGA set were further stratified into two sets using age of 65 years. The results indicated that the high-risk group had a relatively poor prognosis for those both above and below 65 years of age (Figure 6(a), *P* < 0.001, and Figure 6(b), *P* = 0.036). Similarly, the high-risk group had lower overall survival in both the female and the male sets (Figures 6(c) and 6(d), *P* = 0.008 and *P* < 0.001). Moreover, we stratified the samples into T1-2 and T3-4 sets, N0 and N1–3 sets, and M0 and M1 sets. Kaplan-Meier analyses indicated that the high-risk group had a poorer prognosis than the low-risk group in the T1-2 and T3-4 set (Figures 6(e) and 6(f), *P* = 0.046 and *P* < 0.001). We also observed similar results in the N0, N1–3, and M0 sets (Figures 6(g)–6(i), *P* = 0.014, *P* = 0.003, and *P* = 0.002). However, there was no significant difference in the M1 set (Figure 6(j), *P* = 0.254).

### 3.4. Comparison between GI-lncRNA Signatures and Previous lncRNA Signatures

**Figure 7:**
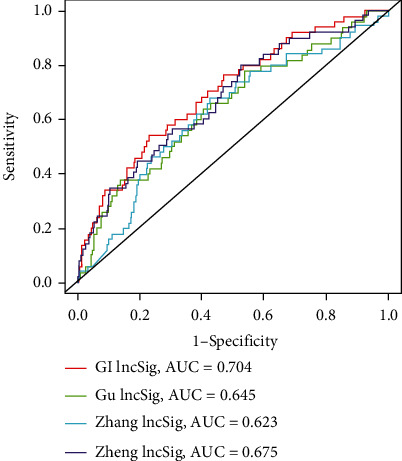
Comparison between the genomic instability-related lncRNA signature and previous lncRNA signatures. Receiver operating characteristic curve analysis of the genomic instability-related lncRNA signature, Gu-lncRNA signature, Cheng-lncRNA signature, and Zhang-lncRNA signature. AUC: area under the curve.

To compare the present prognostic model with the existing lncRNA-related signature, we further performed ROC curve analysis using the same TCGA cohort. Previously published research included a five-lncRNA signature derived from Gu et al. [21], six-lncRNA signature from Cheng et al. [22], and nine-lncRNA signature from Zhang et al. [23]. As a result, the 3-year AUC of the GI-lncRNA signature was 0.704 which was more than the Gu-lncRNA signature (AUC = 0.645), Cheng-lncRNA signature (AUC = 0.675), or Zhang-lncRNA signature (AUC = 0.623) (Figure 7). These results indicated that the GI-lncRNA signature had a better performance in survival prediction.

### 3.5. GI-lncRNA Signature Predicts the Mutation Status of Genes

**Figure 8:**
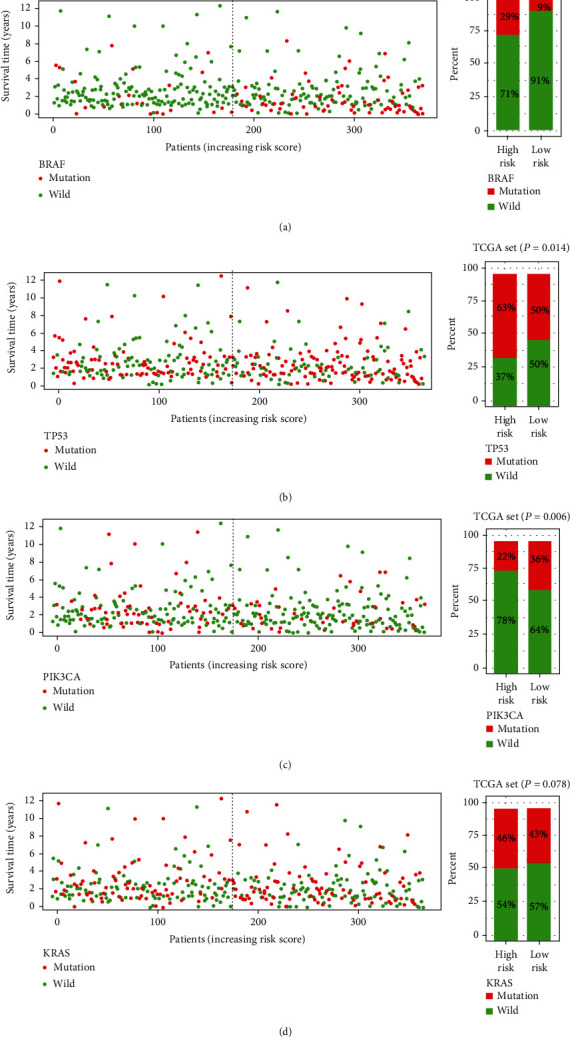
Relationship between risk score and somatic mutation of genes in colorectal cancer. (a) The left figure shows the survival time and BRAF mutation status of patients with increasing risk score. The right figure shows the proportion of BRAF mutation between the high-risk group and the low-risk group in The Cancer Genome Atlas (TCGA) set; (b) the left figure shows the survival time and TP53 mutation status of patients with increasing risk score. The right figure shows the proportion of TP53 mutation between the high-risk and low-risk groups in TCGA set; (c) the left figure shows the survival time and PIK3CA mutation status of patients with increasing risk score; the right figure shows the proportion of PIK3CA mutation between the high-risk and low-risk groups in TCGA set; (d) the left figure shows the survival time and KRAS mutation status of patients with increasing risk score. The right figure shows the proportion of KRAS mutation between the high-risk group and the low-risk group in TCGA set. Red dot or square represents mutation, and green dot or square represents wild-type.

We further analyzed whether the GI-lncRNA signature could predict CRC genetic mutation. We divided all samples into the high-risk and low-risk groups based on the median risk score. The results are shown in Figure 8; the mutation of BRAF (v-Raf murine sarcoma viral oncogene homolog B) and TP53 (tumor protein P53) significantly increased in the high-risk group (Figures 8(a) and 8(b), *P* = 0.008 and *P* = 0.014). However, PIK3CA (phosphatidylinositol-4,5-bisphosphate 3-kinase, catalytic subunit alpha) mutation manifested a higher frequency of mutation in the low-risk group (Figure 8(c), *P* = 0.006). The mutation of KRAS showed no difference between the two groups (Figure 8(d), *P* = 0.708). To a certain extent, the results indicated that the GI-lncRNA signature was able to predict the mutation of BRAF, TP53, and PIK3CA.

## 4. Discussion

Noncoding RNAs including circRNAs [24, 25], miRNAs [26], lncRNAs [27], and circulating tumor DNA [28] have been verified as novel diagnostic biomarkers in malignant tumors. In the present study, we selected lncRNAs as the object for in-depth study. In recent years, targeted therapy and immunotherapy for genomic instability are gradually replacing chemotherapy-based tumor therapy. Scholars have indicated that plentiful mutations produce vast numbers of altered peptides, some of which are expressed and processed as new antigens, to which the immune system can produce antitumor reactions [29]. For CRC patients with microsatellite instability, anti-PD-1 (programmed cell death protein 1) therapy has proven superior to chemotherapy alone in terms of local remission and prognosis [30]. Recently, multiple lncRNAs including lncRNA NORAD [31], lncRNA GUARDIN [32], and BGL3 [33] have been verified to play important roles in genomic instability. Therefore, the construction of the GI-lncRNA signature related to genomic instability has profound implications for CRC diagnosis and treatment.

In the present study, we conducted bioinformatic analysis and identified a total of 143 GI-lncRNAs. However, there is still little research into the role of GI-lncRNAs in CRC. We performed cluster detection to regroup the samples into a GU-like group and a GS-like group. We found that CDX2, MLH1, and PMS2 were expressed at significantly lower levels in the GU-like group. CDX2 has been verified to be a critical biomarker of normal epithelium and prognosis in CRC patients [34, 35]. Furthermore, CDX2 has been indicated to be associated with BRAF mutation and MSI status [34]. MLH1 and PMS2 comprise an important and common mismatch repair protein heterodimer. Salem et al. demonstrated that MLH1/PMS2 loss in CRC has a higher tumor mutation burden than MLH1/PMS2 loss in endometrial cancer [36]. Interestingly, there was no difference in the expression of MSH2 or MSH6 between the GU-like group and the GS-like group. We speculated that the interaction and regulation between MSH2/MSH6 and GI-lncRNAs are relatively weak. Moreover, we found that EGFR was relatively upregulated in the GU-like group. EGFR is a key target in CRC treatment, and studies have indicated that lncRNA SLCO4A1-AS1 [37], lncRNA SCARNA2 [38], lncRNA EGFR-AS1 [39], lncRNA DNAJC3-AS1 [40], and lncRNA LOXL1-AS1 [41] all target EGFR in CRC. LOXL1-AS1 was identified as a GI-lncRNA in the present research.

We conducted GO and KEGG analyses to uncover the biological function of GI-lncRNAs. The results revealed multiple enriched terms related to immunoregulation, genomic instability, and chemokine activity. For instance, incubation of colon cells with IL-6 (interleukin-6) engendered migration of MSH3 from the nucleus to the cytosol and promoted MSI [42]. Wunderlich et al. indicated that IL-6 promoted lymphocyte recruitment through the CCL-20 (CC-chemokine-ligand-20)/CCR-6 (CC-chemokine-receptor-6) axis in the CRC microenvironment [43]. The response to retinoic acid [44] and interferon-gamma [45] was also related to MSI in CRC. Moreover, the enriched terms including leukocyte chemotaxis [46], lymphocyte chemotaxis [47], chemokine activity [48], NOD-like receptor signaling pathway [49], IL-17 signaling pathway [50], Wnt signaling pathway [51], and TNF signaling pathway [52] also play critical roles in CRC.

Furthermore, we performed Cox proportional hazard regression and identified a five-GI-lncRNA signature (PTPRD-AS1, AC009237.14, LINC00543, AP003555.1, and AL109615.3). PTPRD-AS1 has been identified as an immune-related biomarker which predicts overall survival and immunotherapeutic response in bladder cancer [53]. AC009237.14 [22] and AL109615.3 [54] were recently verified as biomarkers based on TCGA database in CRC and gastric cancer, respectively. However, little is known about the GI-lncRNA signature in the progression and genomic instability of CRC. According to the expression of the five-GI-lncRNA signature, we divided the samples into a high-risk and a low-risk group. We found that the signature suggested a difference in prognosis in diverse pathological characteristics except the metastatic set. We compared the AUC value of the GI-lncRNA signature to previously published prognostic signatures via literature review [21–23]. We found that the GI-lncRNA signature obtained the highest AUC value with the lowest number of biomarkers.

A wide array of studies has demonstrated that genetic mutation influences the drug sensitivity and biological behavior of tumors [55]. In CRC, the mutation patterns of BRAF, KRAS, TP53, and PIK3CA are increasingly important in the selection of optimal treatment [56, 57]. In the present research, we found that the five-GI-lncRNA signature captured the mutation status of BRAF, TP53, and PIK3CA. Esposito et al. indicated that silencing of lncRNA COMET increased the drug sensitivity of vemurafenib in BRAF-mutated papillary thyroid cancer [58]. Zhao et al. indicated that expressions of lnc273-31 and lnc273-34 were both elevated in CRC cancer samples with p53-R273H mutation [59]. However, to the best of our knowledge, there was still a lack of lncRNA biomarkers in BRAF and PIK3CA mutation prediction.

The present research is mainly based on bioinformatic analysis and still has some limitations. Firstly, chromosomal instability and MSI have been revealed to have a critical role in genomic instability, but the simple nucleotide variation data could only supply information on mutant genes. Secondly, more molecular biological experiments are needed to verify the identified biomarkers and mechanisms involved in the future.

## 5. Conclusions

Our study provided a bioinformatic strategy to identify lncRNAs and potential mechanisms based on TCGA database and bioinformatic software. Moreover, we identified the five-GI-lncRNA signature as an independent prognostic marker in different cohorts. This GI-lncRNA signature has profound significance in genomic instability and certain value for further research.

## Figures and Tables

**Table 1:** Clinicopathologic characteristics between the train and test groups.

Clinicopathologic characteristics	Type	Train	Test	*P* value
Age (years)	≤65	108 (42.19%)	115 (45.45%)	0.5135
	>65	148 (57.81%)	138 (54.55%)	
Gender	Female	119 (46.48%)	113 (44.66%)	0.7465
	Male	137 (53.52%)	140 (55.34%)	
Stage	Stage I-II	134 (52.34%)	145 (57.31%)	0.3641
	Stage III-IV	113 (44.14%)	102 (40.32%)	
	Unknown	9 (3.52%)	6 (2.37%)	
T	T1-2	51 (19.92%)	53 (20.95%)	0.8593
	T3-4	205 (80.08%)	200 (79.05%)	
M	M0	185 (72.27%)	195 (77.08%)	0.1698
	M1	42 (16.41%)	30 (11.86%)	
	Unknown	29 (11.33%)	28 (11.07%)	
N	N0	145 (56.64%)	151 (59.68%)	0.5095
	N1-3	111 (43.36%)	101 (39.92%)	
	Unknown	0 (0%)	1 (0.4%)	

**Table 2:** Cox regression analyses of 5-GI-lncRNA signature in TCGA set.

Variable	Univariate analysis			Multivariate analysis	
	HR (95% CI)	*P* value	Coefficient	HR (95% CI)	*P* value
AP003555.1 (high/low)	1.343 (1.171-1.538)	<0.001^∗∗^	0.469	1.598 (1.267-2.014)	<0.001^∗∗^
AC009237.14 (high/low)	1.170 (1.077-1.271)	<0.001^∗∗^	0.182	1.199 (1.078-1.334)	<0.001^∗∗^
AL109615.3 (high/low)	1.070 (1.016-1.126)	0.015^∗^	0.072	1.074 (1.022-1.130)	0.005^∗∗^
LINC00543 (high/low)	1.046 (1.008-1.109)	0.047^∗^	0.063	1.065 (1.005-1.129)	0.033^∗^
PTPRD-AS1 (high/low)	0.645 (0.442-0.943)	0.024^∗^	-0.447	0.639 (0.436-0.937)	0.022^∗^

^∗^ *P* < 0.05,  ^∗∗^*P* < 0.01.

**Table 3:** Cox regression analyses of pathological characteristics and risk score.

Variable	Univariate analysis		Multivariate analysis	
	HR (95% CI)	*P* value	HR (95% CI)	*P* value
Age (years)	1.028 (1.009-1.047)	0.004^∗∗^	1.033 (1.012-1.049)	0.003^∗∗^
Gender (male/female)	1.095 (0.728-1.646)	0.664		
pT stage (T1/T2/T3/T4)	2.927 (1.948-4.396)	<0.001^∗∗^	2.079 (1.339-3.226)	<0.001^∗∗^
pN stage (N0/N1/N2)	2.901 (1.898-4.434)	<0.001^∗∗^	1.692 (1.025-2.795)	0.039^∗^
pM stage (M0/M1)	4.753 (3.107-7.269)	<0.001^∗∗^	2.989 (1.799-4.966)	<0.001^∗∗^
Risk score (high/low)	1.248 (1.131-1.378)	<0.001^∗∗^	1.056 (1.021-1.092)	<0.001^∗∗^

^∗^ *P* < 0.05,  ^∗∗^*P* < 0.01.

## Data Availability

The raw data of this study are derived from TCGA database (https://portal.gdc.cancer.gov/), which is a publicly available database.

## Supplementary Materials

Supplementary Materials Supplementary Figure 1: cluster detection of 568 colorectal cancer samples. The patients were divided into a GS-like group and a GU-like group based on the expression of 143 genomically unstable-related lncRNAs. Supplementary Figure 2: coexpression network diagram of genomic instability-related lncRNAs and corresponding mRNAs. Green dot represents lncRNA, and red dot represents mRNA. Supplementary Material: list of GI-lncRNAs.
