# Supplementary material for: Identification of a Genomic Instability-Related Long Noncoding RNA Prognostic Model in Colorectal Cancer Based on Bioinformatic Analysis
Source: Dis Markers. 2022 Jun 7;2022:4556585. doi: 10.1155/2022/4556585 (PMC9197617; doi:10.1155/2022/4556585)
Supplement: Supplementary Materials — Supplementary Figure 1: cluster detection of 568 colorectal cancer samples. The patients were divided into a GS-like group and a GU-like group based on the expression of 143 genomically unstable-related lncRNAs. Supplementary Figure 2: coexpression network diagram of genomic instability-related lncRNAs and corresponding mRNAs. Green dot represents lncRNA, and red dot represents mRNA. Supplementary Material: list of GI-lncRNAs. [file 4556585.f1.docx]

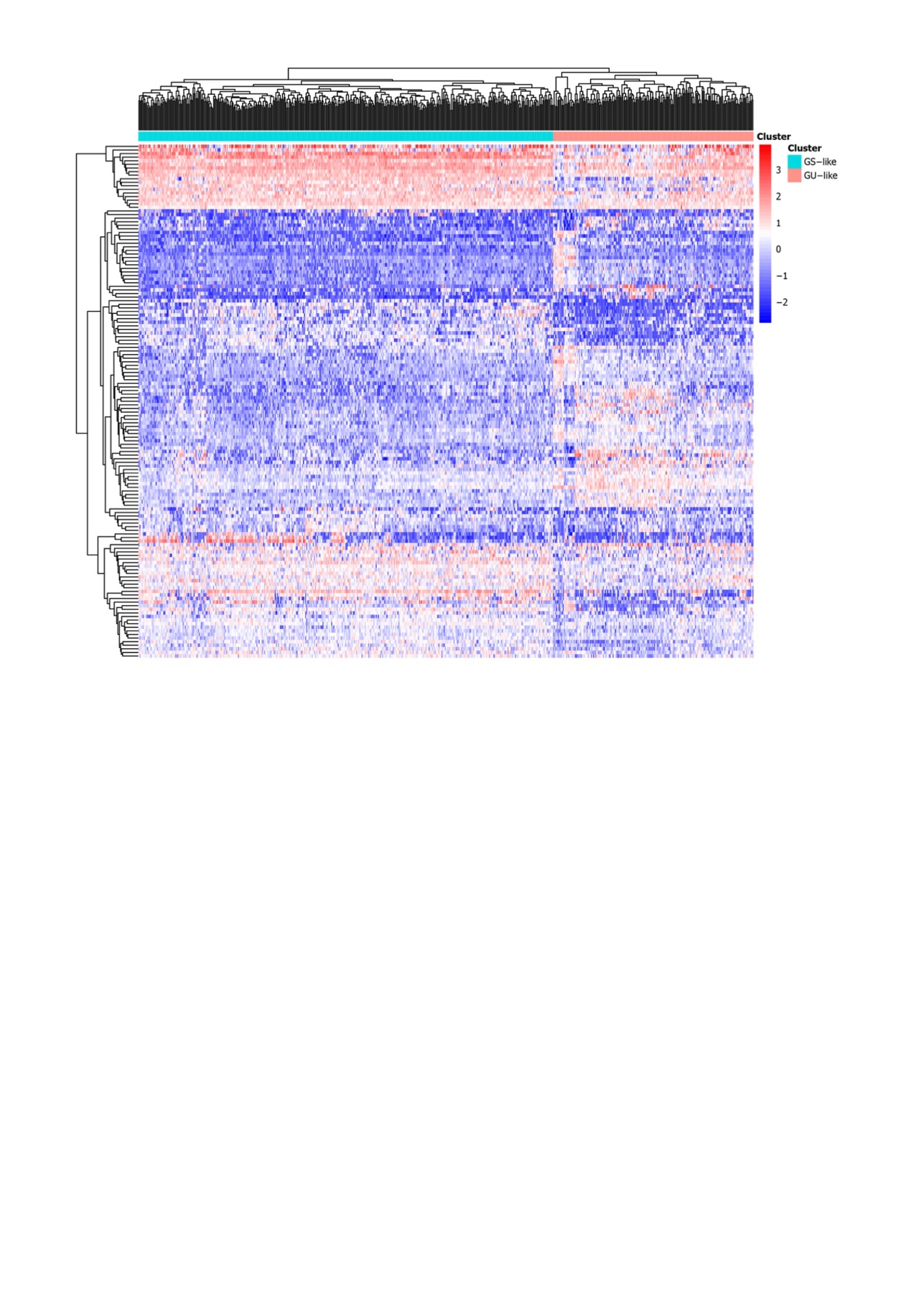


**Supplementary Figure 1. Cluster detection of 568 colorectal cancer samples.** The patients were divided into a GS-like group and a GU-like group based on the expression of 143 genomically-unstable-related lncRNAs.


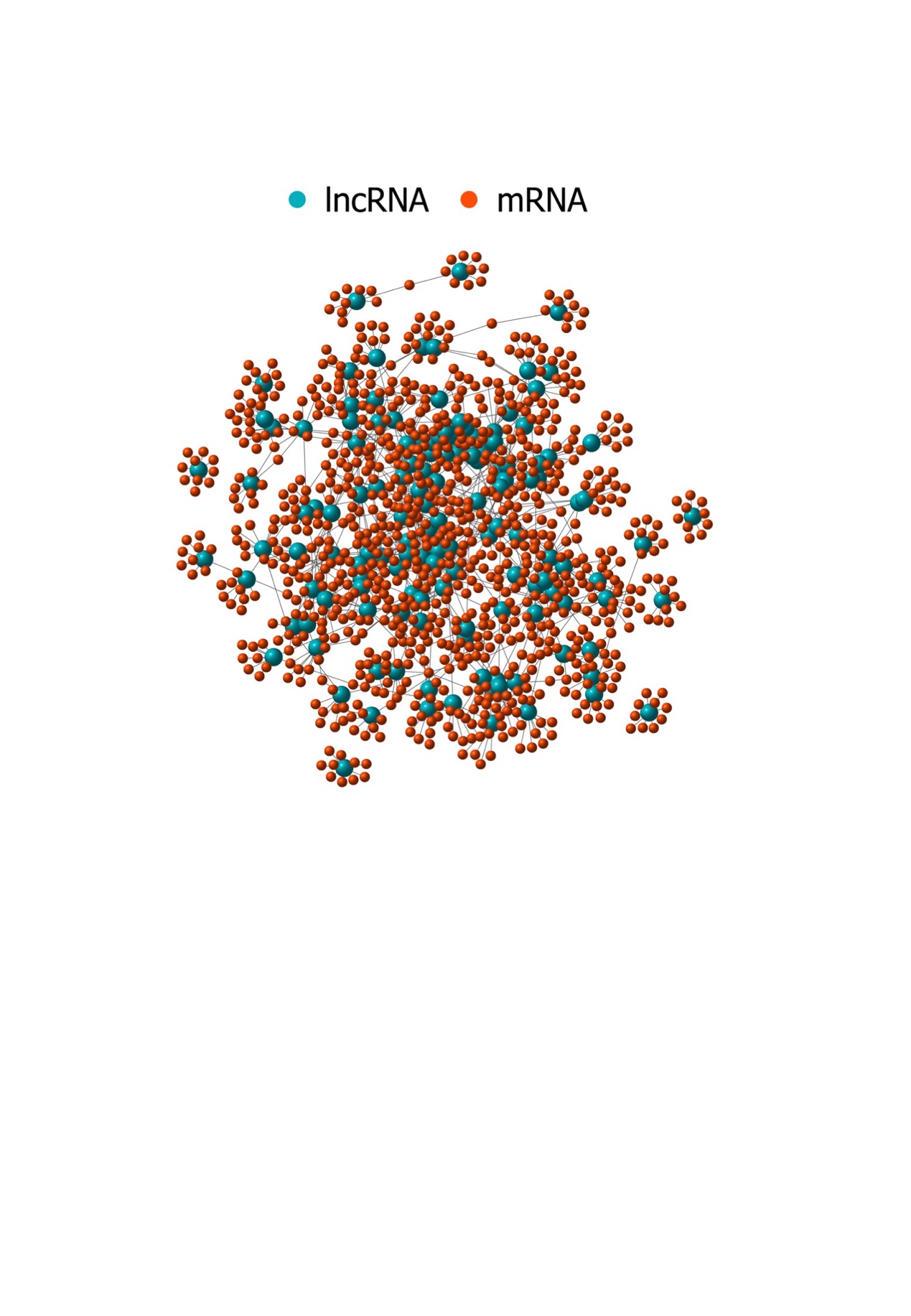


**Supplementary Figure 2. Co-expression network diagram of genomic instability-related lncRNAs and corresponding mRNAs.** Green dot represents lncRNA and red dot represents mRNA.

Supplementary Material: list of GI-lncRNAs

| GI-lncRNA | GS sample | GU samples | logFC | *p*-Value |
| --- | --- | --- | --- | --- |
| AC136475.9 | 0.773 | 0.125 | -2.628 | 6.950E-09 |
| AC026336.3 | 1.375 | 0.223 | -2.624 | 7.448E-12 |
| AC135388.1 | 0.704 | 0.135 | -2.388 | 5.214E-13 |
| AC004231.1 | 0.688 | 0.140 | -2.293 | 1.088E-17 |
| AC078993.1 | 4.120 | 0.919 | -2.165 | 7.087E-13 |
| AC110772.2 | 1.183 | 0.272 | -2.119 | 2.380E-04 |
| AP003774.2 | 17.033 | 4.133 | -2.043 | 3.423E-17 |
| AC108865.1 | 7.175 | 1.840 | -1.963 | 1.327E-10 |
| AC108865.2 | 2.790 | 0.731 | -1.933 | 9.353E-08 |
| AC010280.1 | 0.948 | 0.258 | -1.880 | 1.476E-11 |
| AC114296.1 | 0.870 | 0.244 | -1.837 | 1.280E-12 |
| LINC00654 | 1.574 | 0.442 | -1.833 | 6.863E-14 |
| LINC01811 | 0.914 | 0.269 | -1.766 | 5.329E-08 |
| AC093585.1 | 0.680 | 0.215 | -1.663 | 3.860E-11 |
| TUSC8 | 3.985 | 1.272 | -1.648 | 3.631E-17 |
| LINC00525 | 0.827 | 0.268 | -1.626 | 8.083E-17 |
| BOK-AS1 | 1.790 | 0.597 | -1.584 | 2.619E-03 |
| LINC01082 | 1.895 | 0.643 | -1.561 | 2.688E-15 |
| PTPRD-AS1 | 1.017 | 0.354 | -1.524 | 2.874E-15 |
| AC108134.3 | 2.841 | 0.988 | -1.524 | 1.581E-12 |
| AL390208.1 | 0.681 | 0.241 | -1.501 | 9.516E-03 |
| AL365226.1 | 44.615 | 16.019 | -1.478 | 1.087E-07 |
| AL117382.2 | 12.624 | 4.721 | -1.419 | 3.098E-17 |
| AP001554.1 | 0.637 | 0.239 | -1.415 | 4.733E-08 |
| AC064807.2 | 0.965 | 0.367 | -1.393 | 2.627E-07 |
| CHN2-AS1 | 0.786 | 0.302 | -1.382 | 1.059E-09 |
| AC055717.2 | 1.758 | 0.684 | -1.363 | 1.448E-12 |
| LINC02441 | 3.516 | 1.382 | -1.347 | 9.240E-15 |
| AC254629.1 | 5.319 | 2.136 | -1.316 | 2.537E-16 |
| AL162582.1 | 2.011 | 0.814 | -1.305 | 2.183E-07 |
| AC009812.1 | 1.423 | 0.578 | -1.299 | 6.606E-15 |
| DPP10-AS1 | 1.130 | 0.465 | -1.280 | 5.853E-06 |
| HNF4A-AS1 | 1.205 | 0.498 | -1.276 | 3.571E-03 |
| AP005271.1 | 0.769 | 0.319 | -1.269 | 5.747E-09 |
| AL121832.1 | 1.102 | 0.458 | -1.267 | 4.001E-09 |
| AC022613.1 | 1.118 | 0.465 | -1.266 | 3.589E-11 |
| AL121829.2 | 0.747 | 0.313 | -1.255 | 9.948E-07 |
| SATB2-AS1 | 3.236 | 1.357 | -1.254 | 7.249E-14 |
| AL022313.2 | 1.085 | 0.455 | -1.254 | 2.015E-08 |
| AL355312.3 | 0.931 | 0.392 | -1.249 | 9.870E-06 |
| RARA-AS1 | 3.545 | 1.513 | -1.229 | 9.464E-07 |
| AL133346.1 | 0.579 | 0.247 | -1.228 | 8.124E-10 |
| LINC02487 | 1.365 | 0.583 | -1.228 | 1.821E-14 |
| AL390198.1 | 3.221 | 1.378 | -1.225 | 4.426E-08 |
| ZNF503-AS1 | 1.895 | 0.811 | -1.224 | 1.036E-07 |
| RHPN1-AS1 | 1.081 | 0.464 | -1.221 | 7.103E-12 |
| DIO3OS | 3.952 | 1.700 | -1.217 | 5.664E-12 |
| AC017074.1 | 3.118 | 1.343 | -1.215 | 2.438E-17 |
| SMIM2-AS1 | 1.894 | 0.817 | -1.212 | 5.553E-09 |
| AP000785.1 | 0.648 | 0.281 | -1.208 | 1.842E-07 |
| OSER1-DT | 5.399 | 2.343 | -1.204 | 7.502E-21 |
| AC026801.2 | 0.716 | 0.311 | -1.203 | 5.896E-14 |
| LINC02747 | 7.665 | 3.341 | -1.198 | 6.941E-16 |
| AL139384.1 | 0.858 | 0.377 | -1.187 | 1.325E-11 |
| AC080129.2 | 1.329 | 0.590 | -1.171 | 4.724E-11 |
| LINC01558 | 1.984 | 0.883 | -1.168 | 1.192E-10 |
| AC053503.3 | 2.435 | 1.087 | -1.164 | 2.519E-06 |
| AL133370.1 | 8.704 | 3.899 | -1.159 | 4.837E-06 |
| AC106876.1 | 7.850 | 3.523 | -1.156 | 1.018E-17 |
| AL391056.1 | 1.631 | 0.738 | -1.145 | 2.737E-11 |
| AC090579.1 | 0.777 | 0.352 | -1.143 | 4.461E-13 |
| LINC02563 | 0.789 | 0.359 | -1.136 | 5.317E-08 |
| LINC02418 | 4.635 | 2.113 | -1.133 | 6.364E-11 |
| AP004608.1 | 1.311 | 0.598 | -1.132 | 1.872E-11 |
| AC009061.1 | 0.634 | 0.290 | -1.130 | 1.202E-04 |
| LHFPL3-AS2 | 2.745 | 1.280 | -1.100 | 3.451E-04 |
| LINC00543 | 3.695 | 1.733 | -1.093 | 5.485E-16 |
| LINC01315 | 5.096 | 2.417 | -1.076 | 3.949E-13 |
| AL121895.2 | 0.788 | 0.376 | -1.066 | 9.210E-07 |
| LINC00653 | 0.790 | 0.379 | -1.060 | 1.063E-08 |
| AC009237.14 | 2.225 | 1.070 | -1.057 | 1.199E-10 |
| AC109446.3 | 1.881 | 0.906 | -1.054 | 1.681E-11 |
| AC123023.1 | 1.903 | 0.917 | -1.053 | 8.884E-11 |
| LINC01807 | 0.636 | 0.309 | -1.039 | 3.239E-04 |
| AC004233.2 | 3.970 | 1.941 | -1.033 | 8.832E-10 |
| CASC19 | 5.391 | 2.695 | -1.000 | 2.531E-05 |
| LINC01094 | 0.388 | 0.778 | 1.005 | 2.944E-06 |
| AC106037.2 | 0.285 | 0.580 | 1.027 | 1.752E-09 |
| C1orf195 | 0.420 | 0.859 | 1.034 | 1.014E-02 |
| AC005632.3 | 0.320 | 0.666 | 1.059 | 2.650E-08 |
| LOXL1-AS1 | 0.559 | 1.170 | 1.066 | 4.105E-09 |
| AC087286.2 | 0.409 | 0.859 | 1.072 | 2.592E-02 |
| AC027514.2 | 0.273 | 0.576 | 1.076 | 2.023E-03 |
| AC026202.2 | 0.277 | 0.584 | 1.076 | 6.383E-03 |
| LINC02100 | 0.338 | 0.716 | 1.082 | 8.021E-03 |
| AC253536.3 | 0.496 | 1.052 | 1.085 | 1.337E-03 |
| AP001107.4 | 0.328 | 0.700 | 1.093 | 2.078E-04 |
| AC023043.4 | 0.699 | 1.501 | 1.103 | 1.595E-16 |
| NPTN-IT1 | 0.470 | 1.017 | 1.113 | 1.740E-03 |
| AC018410.1 | 0.284 | 0.622 | 1.132 | 3.271E-03 |
| AGAP1-IT1 | 0.814 | 1.819 | 1.161 | 7.392E-08 |
| AL109615.3 | 0.584 | 1.313 | 1.168 | 6.691E-08 |
| AL132989.2 | 0.321 | 0.724 | 1.175 | 3.390E-08 |
| AL136115.2 | 0.560 | 1.271 | 1.183 | 2.409E-03 |
| LINC02688 | 0.372 | 0.849 | 1.189 | 1.404E-06 |
| AC091588.1 | 0.519 | 1.186 | 1.194 | 2.077E-04 |
| AC005911.1 | 0.399 | 0.916 | 1.199 | 3.461E-11 |
| AC092756.1 | 0.280 | 0.646 | 1.208 | 1.427E-02 |
| AC115522.1 | 0.382 | 0.885 | 1.211 | 4.080E-09 |
| AC009163.6 | 0.296 | 0.688 | 1.216 | 6.552E-04 |
| AC005392.2 | 1.889 | 4.488 | 1.249 | 4.131E-04 |
| AC007991.2 | 0.821 | 1.959 | 1.254 | 4.141E-04 |
| AP003555.1 | 0.336 | 0.800 | 1.254 | 1.215E-04 |
| MIAT | 0.465 | 1.116 | 1.262 | 2.089E-06 |
| AC012317.1 | 0.482 | 1.170 | 1.279 | 8.172E-06 |
| MIR924HG | 0.330 | 0.801 | 1.281 | 1.016E-12 |
| AC112484.3 | 0.264 | 0.650 | 1.299 | 6.305E-04 |
| AC007996.1 | 0.520 | 1.296 | 1.316 | 1.031E-15 |
| AC090181.2 | 1.281 | 3.192 | 1.317 | 5.840E-10 |
| AL606834.1 | 0.555 | 1.397 | 1.332 | 1.343E-09 |
| AC023825.2 | 0.225 | 0.572 | 1.346 | 2.254E-05 |
| MAP3K5-AS1 | 0.360 | 0.951 | 1.400 | 9.000E-05 |
| AC005256.1 | 0.578 | 1.526 | 1.401 | 1.003E-06 |
| LINC02041 | 0.714 | 1.904 | 1.415 | 1.800E-09 |
| AC104695.4 | 1.271 | 3.517 | 1.468 | 2.424E-03 |
| LINC02826 | 0.424 | 1.180 | 1.477 | 4.185E-09 |
| AC118754.1 | 0.223 | 0.626 | 1.491 | 4.827E-03 |
| LIMS1-AS1 | 0.233 | 0.665 | 1.514 | 7.217E-04 |
| AP005899.1 | 0.415 | 1.192 | 1.522 | 6.662E-09 |
| LINC00941 | 0.391 | 1.159 | 1.566 | 1.880E-09 |
| MADD-AS1 | 0.203 | 0.613 | 1.590 | 1.288E-03 |
| AP002498.1 | 1.061 | 3.289 | 1.633 | 1.584E-02 |
| LINC02489 | 0.477 | 1.496 | 1.648 | 2.300E-07 |
| AL157871.2 | 0.296 | 0.944 | 1.672 | 9.870E-08 |
| AL138789.1 | 0.199 | 0.639 | 1.682 | 1.121E-04 |
| LINC01443 | 0.205 | 0.660 | 1.684 | 7.537E-05 |
| LINC02195 | 0.339 | 1.092 | 1.686 | 8.652E-14 |
| AL121761.1 | 0.614 | 2.034 | 1.729 | 1.291E-04 |
| AC114760.2 | 0.220 | 0.750 | 1.766 | 1.282E-05 |
| UNC5B-AS1 | 0.421 | 1.440 | 1.773 | 1.811E-05 |
| HIF1A-AS3 | 0.691 | 2.409 | 1.801 | 1.409E-03 |
| AC245128.3 | 0.239 | 0.834 | 1.806 | 7.623E-04 |
| TFAP2A-AS1 | 0.294 | 1.043 | 1.826 | 7.036E-16 |
| AL022316.1 | 0.441 | 1.808 | 2.036 | 6.324E-09 |
| AC010998.3 | 0.214 | 0.925 | 2.112 | 1.753E-04 |
| AC106037.3 | 0.171 | 0.785 | 2.201 | 9.621E-04 |
| AC022784.1 | 0.170 | 0.900 | 2.406 | 1.982E-10 |
| AFAP1-AS1 | 0.875 | 4.661 | 2.413 | 3.620E-04 |
| AC092868.3 | 0.292 | 1.589 | 2.442 | 3.340E-03 |
| AC036176.3 | 0.128 | 0.716 | 2.481 | 3.696E-04 |
| LINC02446 | 0.205 | 1.264 | 2.627 | 6.468E-15 |
| AC092112.1 | 0.174 | 1.254 | 2.848 | 1.358E-03 |
| DLGAP1-AS5 | 0.028 | 1.282 | 5.498 | 7.658E-07 |
